# Potential Implementers’ Perspectives on the Development and Implementation of an e–Mental Health Intervention for Caregivers of Adults With Chronic Kidney Disease: Qualitative Interview Study

**DOI:** 10.2196/51461

**Published:** 2023-11-17

**Authors:** Chelsea Coumoundouros, Rabie Adel El Arab, Paul Farrand, Alexander Hamilton, Robbert Sanderman, Louise von Essen, Joanne Woodford

**Affiliations:** 1 Healthcare Sciences and e-Health Department of Women’s and Children’s Health Uppsala University Uppsala Sweden; 2 Clinical Education, Development and Research (CEDAR), Psychology University of Exeter Exeter United Kingdom; 3 Faculty of Nursing and Physiotherapy University of Lleida Lleida Spain; 4 Healthcare Research Group (GRECS) Institute for Biomedical Research (IRBLleida) Lleida Spain; 5 Exeter Kidney Unit Royal Devon University Healthcare NHS Foundation Trust Exeter United Kingdom; 6 Faculty of Health and Life Sciences University of Exeter Medical School University of Exeter Exeter United Kingdom; 7 Department of Health Psychology University Medical Center Groningen University of Groningen Groningen Netherlands

**Keywords:** health care professional, implementation, informal caregiver, chronic kidney disease, e–mental health, Consolidated Framework for Implementation Research

## Abstract

**Background:**

e–Mental health interventions can improve access to mental health support for caregivers of people living with chronic kidney disease (CKD). However, implementation challenges often prevent effective interventions from being put into practice. To develop an e–mental health intervention for caregivers of people living with CKD that is optimized for future implementation, it is important to engage professionals that may endorse or deliver the intervention (ie, potential implementers) during intervention development.

**Objective:**

This study aims to explore the perspectives of potential implementers working in kidney care, in mental health care, or at nonprofit organizations regarding the design and implementation of an e–mental health intervention for caregivers of people living with CKD.

**Methods:**

Potential implementers (N=18) were recruited via National Health Service Trusts, email, and social media advertisements to participate in semistructured video interviews. Interview questions were informed by the Consolidated Framework for Implementation Research (CFIR). Data were analyzed using a deductive analysis approach using the CFIR, with inductive coding applied to relevant data not captured by the framework.

**Results:**

A total of 29 generic categories, related to 17 CFIR constructs, were identified. The perceived fit between the intervention and implementation context (ie, existing service delivery models and work routines) and existing social networks among potential implementers were perceived as important factors in enhancing implementation potential. However, a need for capacity building among potential implementers to create systems to support the identification and referral of caregivers to an e–mental health intervention was identified. Equity concerns were raised regarding the intervention, highlighting the importance of incorporating an equity lens during intervention design to enhance accessibility and adoption.

**Conclusions:**

Potential implementers provided valuable insights into key design and implementation factors to help inform the development of an e–mental health intervention for caregivers of people living with CKD. Incorporating their feedback can help ensure the intervention is acceptable and inform the selection of future implementation strategies to enhance the implementation potential of the intervention. Potential implementers should continue to be engaged throughout intervention development.

## Introduction

### Background

Caregivers (ie, family and friends who provide unpaid care to someone living with a physical or mental health condition) commonly experience mental health problems such as depression and anxiety [1,2]. However, few access mental health support [3,4]. Barriers to accessing mental health support for caregivers include lacking the time needed to attend face-to-face appointments, experiencing guilt for focusing on their own needs, and not prioritizing time to focus on their own mental and physical health [5]. Delivering mental health interventions via e–mental health has significant potential to improve access to mental health support for caregivers [6]. For example, internet delivery may alleviate barriers to access given that e–mental health interventions can be accessed at any time without needing to travel to attend appointments and may enhance anonymity [6].

### The Implementation of e–Mental Health Interventions for Caregivers

Despite evidence suggesting e–mental health interventions can be effective for caregivers [7,8], implementation challenges commonly prevent adoption into routine health care practice. An evaluation of 12 eHealth and e–mental health interventions developed for caregivers of people with dementia indicated interventions were generally not *implementation ready*, with little information available concerning important factors required for implementation, such as staffing and training resources [9]. A recent systematic review of the implementation of e–mental health interventions for caregivers of adults with chronic diseases identified that factors related to the implementation setting and wider context (eg, available resources, relative priority of the intervention, and external policies) have been largely neglected [10]. In addition, professionals (eg, potential implementers) were seldom engaged in understanding how interventions would fit within the current health care practice [10]. Therefore, research suggests that existing e–mental health interventions have low implementation potential, limiting intervention adoption and long-term sustainability [11].

To optimize the implementation potential of e–mental health interventions, factors that may influence implementation should be considered during intervention development [11,12]. In the new Medical Research Council (MRC) complex interventions framework, understanding key contextual factors, including the implementation setting, and engaging key stakeholders during intervention development, testing, and evaluation phases is recommended [11]. Intervention development studies have started to apply this approach by engaging with stakeholders to explore implementation while developing interventions [13,14]. Stakeholder involvement may enhance our understanding of how organizations can support future intervention implementation, what barriers and facilitators to implementation exist to inform future implementation strategies, and how to best deliver an intervention within existing practice [13,14].

### Tailoring Interventions for Caregivers of People Living With Chronic Kidney Disease

Caregivers of people living with chronic kidney disease (CKD) are often neglected in existing research [15-17]. Despite depression and anxiety being commonly reported [18,19], few mental health interventions have been tailored for this population [17]. Tailoring interventions can enhance acceptability [20,21] and ensure intervention content meets the needs and preferences of caregivers of people living with CKD [10]. Given the current lack of tailored support, we aimed to develop an e–mental health intervention, optimized for future implementation, for caregivers of people living with CKD by using the new MRC framework [11] and intervention development framework [12]. Within this study, select core elements within the MRC framework (considering context, engaging stakeholders, and identifying key uncertainties) [11] were addressed, and select actions of the intervention development framework (undertake primary data analysis, understand context, and pay attention to future implementation of the intervention in the real world) [12] were used to support a theory- and evidence-based approach to the initial development of an e–mental health intervention for caregivers of people living with CKD [22].

### Research Aim

We aimed to explore the perspectives of professionals (ie, potential implementers) anticipated to play key roles in the future implementation of an e–mental health intervention for caregivers of people living with CKD regarding the intervention’s design, delivery, and implementation.

## Methods

### Study Design

We conducted a qualitative description study [23] using semistructured interviews with the analysis remaining close to the manifest content. Pragmatism was adopted as the overall research paradigm, selecting the methods that best suited the goal of this research (ie, professional stakeholder perspectives on intervention design, delivery, and implementation) [24]. The results are reported following the Standards for Reporting Qualitative Research [25] (Multimedia Appendix 1).

### Ethical Considerations

Ethics approval to interview professionals working for the National Health Service (NHS) was obtained from the University of Exeter Psychology Research Ethics Committee (reference: 510971) and from the Health Research Authority (Integrated Research Application System number: 308682). Ethics approval to interview professionals at nonprofit organizations was obtained from the University of Exeter Psychology Research Ethics Committee (reference: 513911). As some research team members are based in Sweden, ethics approval to conduct remote data collection and analysis from Sweden was obtained from the Swedish Ethical Review Authority (dnr: 2022-03068-01). Written informed consent was obtained from all participants before data collection, and consent was verbally reaffirmed immediately before beginning each interview.

### Context

Participants could be located anywhere in the United Kingdom. Within the United Kingdom, mental health support for caregivers of people living with CKD could potentially be provided by the NHS Talking Therapies for Anxiety and Depression service (formerly known as Improving Access to Psychological Therapies [26]), kidney care units, or nonprofit organizations for caregivers (including general caregiver organizations and CKD specific organizations). Professionals working in each setting could potentially be involved in future implementation, that is, endorsement or delivery of the e–mental health intervention.

### Sampling

A variation sampling technique [27] was adopted to purposefully sample professionals working within each setting (ie, kidney care, mental health care, and nonprofit organizations) where implementation could occur. Health care professionals (HCPs) working in mental or kidney health care were recruited primarily through 4 NHS Trusts in the South West of England via email; however, HCPs working for any NHS Trust were eligible to participate. Study advertisements were also shared via social media, professional networks, and word of mouth. Professionals working at nonprofit organizations were contacted directly via email by the research team with a study advertisement. Interested professionals were provided with a participant information sheet, a consent form, and an opportunity to ask questions.

### Data Collection

Semistructured interviews were conducted by CC via video call between May 2022 and January 2023 and recorded on an external audio recorder. In total, 18 interviews were conducted, ranging from 40 to 110 minutes, with a mean length of 58 minutes (SD 18 min). After providing informed consent, professionals were given a brief written description of the proposed e–mental health intervention (Multimedia Appendix 2), described as a cognitive behavioral therapy (CBT)–based internet-administered intervention that may be supported by a trained professional. A CBT-based intervention was proposed given that internet-administered CBT is effective for depression and anxiety [7,28] and that CBT is the predominant therapeutic method adopted by the NHS Talking Therapies for Anxiety and Depression service [29].

Professionals typically had 1 to 2 weeks to review the intervention description before the interview. An interview guide, partly informed by the Consolidated Framework for Implementation Research (CFIR) [30], was followed, exploring professionals’ perspectives on the design, delivery, and implementation of the e–mental health intervention (Multimedia Appendix 2). The CFIR is an implementation framework that outlines factors that can influence implementation related to 5 domains: *innovation* (ie, the intervention being implemented); *inner setting* (ie, the setting in which the intervention is being implemented); *outer setting* (ie, the setting in which the inner setting exists, including the health care system, community, the state); *individuals* (ie, the roles and characteristics of individuals who may implement or engage with the intervention, partly based on the Capability, Opportunity, Motivation–Behavior system [31]); and *implementation process* (ie, activities and strategies used to implement the intervention) [32]. The questions explored topics such as intervention-workplace fit, what evidence about the intervention was desired, barriers and facilitators to intervention use by both potential implementers and caregivers, and potential implementer views of caregivers. All the views reported are from the perspective of potential implementers.

### Sample Characteristics

A total of 18 professionals (n=14, 78% women and n=4, 22% men) with a mean age of 49 (SD 9) years, working in England (n=14) or Wales (n=4) participated. Professionals worked in kidney health care (n=9), in general mental health care (n=3), or at nonprofit organizations (n=6), having worked on average for 7 (SD 5) years in their current role. Kidney HCPs worked in England (n=8) or Wales (n=1) and included a renal dietician, renal nurses, a nephrologist, a renal psychologist, and a renal social worker. The background characteristics of professionals are summarized in Table 1, with individual-level characteristics available in Multimedia Appendix 3.

**Table 1:** Background characteristics of potential implementers (N=18).

Characteristics				Values
**General characteristics**				
	Age (years), mean (SD)		49 (9)	
	**Gender,** **n (%)**			
		Women	14 (78)	
		Men	4 (22)	
	Time in current role (years), mean (SD)		7 (5)	
	Working in England, n (%)		14 (78)	
**Role, n (%)**				
	Kidney HCP^a^		9 (50)	
	Mental HCP		3 (17)	
	NPO^b^ professional		6 (33)	
**Experience working with specific populations, n (%)**				
	Caregivers of people with CKD^c^		15 (83)	
	People with CKD		15 (83)	
	People with mental health problems		17 (94)	
	Caregivers of people with other chronic diseases		13 (72)	

^a^HCP: health care professional.

^b^NPO: nonprofit organization.

^c^CKD: chronic kidney disease.

### Data Analysis

#### Overview

The interviews were transcribed verbatim by a professional transcription company, with NVivo (QSR International) used to support the analysis. Content analysis [33] was selected given that this approach aligned with the objective of identifying factors to be considered when designing and implementing e–mental health interventions for caregivers. CFIR constructs [32] informed deductive coding, and inductive coding was used to code data relevant to research objectives that did not fit within the CFIR. Data analysis was informed by a similar study using the CFIR to explore implementation determinants [34].

CC read all 18 interview transcripts and RAEA read 7 interview transcripts, recording initial impressions. Initially, 7 transcripts were independently coded by CC and RAEA using a codebook that included all CFIR constructs as codes [32]. CC and RAEA held regular meetings (n=6) to discuss their understanding of the CFIR, ensure the constructs were applied consistently to the data, and critically assess how the framework fit with the data. This resulted in adding a construct to the codebook called *Knowledge and beliefs about the innovation*, as the codebook lacked a construct related to an individual’s beliefs and views of the intervention. This construct was informed by a previous version of the CFIR [30]; however, this construct was removed from the most recent version of the CFIR used for coding [32]. Owing to resource limitations, the remaining 11 transcripts were only coded by CC, with triangulation through dialogue with JW to establish rigor.

Following the coding of data into the appropriate CFIR construct, the data within each construct were inductively organized into generic categories and subcategories [33,34] by CC and reviewed by JW and RAEA. The final category revision was performed by CC. Descriptions of CFIR constructs identified in the data, along with a table of generic categories and subcategories, were provided to PF for peer examination and revised after discussion between CC and JW. Rigor and trustworthiness were established by maintaining an audit trail of meeting minutes and impressions of the data [35] and investigator triangulation [36] by having (1) a second researcher code a portion of transcripts, (2) 2 researchers hold regular meetings to enhance the conceptual understanding of the CFIR and ensure consistent application to the data, and (3) regular dialogue with other research team members with different backgrounds and levels of experience. As a qualitative study, we did not compare the concerns expressed by different professional groups. However, disconfirming cases [37] and divergent views were actively sought [37,38].

#### CFIR Tailoring

Some tailoring of the CFIR codebook was necessary given that the interviews explored the future implementation of a proposed intervention. Given that the exact role professionals would have in the future implementation of the e–mental health intervention was unknown (eg, whether they would be an implementation facilitator or implementation lead), a more generic role of “potential implementer” was created for use within the CFIR domains *individuals* and *implementation process*. The role “potential implementer” refers to all study participants, including roles related to implementing, delivering, or endorsing the intervention. In addition, given that the implementation setting (ie, the *inner setting* domain within the CFIR representing the specific organization in which an intervention is implemented) was undefined and participants worked in a variety of potential implementation settings, distinguishing between the *inner* and *outer setting* domains was difficult. Therefore, we considered CFIR constructs within the *inner* and *outer setting* domains as falling within a single combined *inner/outer setting* domain, reflecting the general implementation context. Multimedia Appendix 4 shows the modifications made to the CFIR and adapted construct definitions.

### Researcher Characteristics

Interviews were conducted by the first author (CC) who also led the analysis. CC is a female PhD candidate with a background in public health. She has experience using the original version of the CFIR [30] for qualitative data analysis and has conducted research related to informal caregivers and e–mental health implementation. CC had no preexisting relationships with any participants. RAEA is a male PhD candidate with a background in nursing and no prior experience using the CFIR. JW is a female researcher with a PhD in psychology and extensive experience in conducting qualitative research. JW is the principal investigator of the study and has been a member of the research team since conception. PF is an expert in CBT self-help interventions, including e–mental health, is a member of the NHS Expert Advisory Group for the NHS Talking Therapies for Anxiety and Depression program and the NICE Medical Technologies Advisory Group, and has extensive experience conducting qualitative research, recently within the renal specialism.

## Results

### Overview

**Figure 1:**
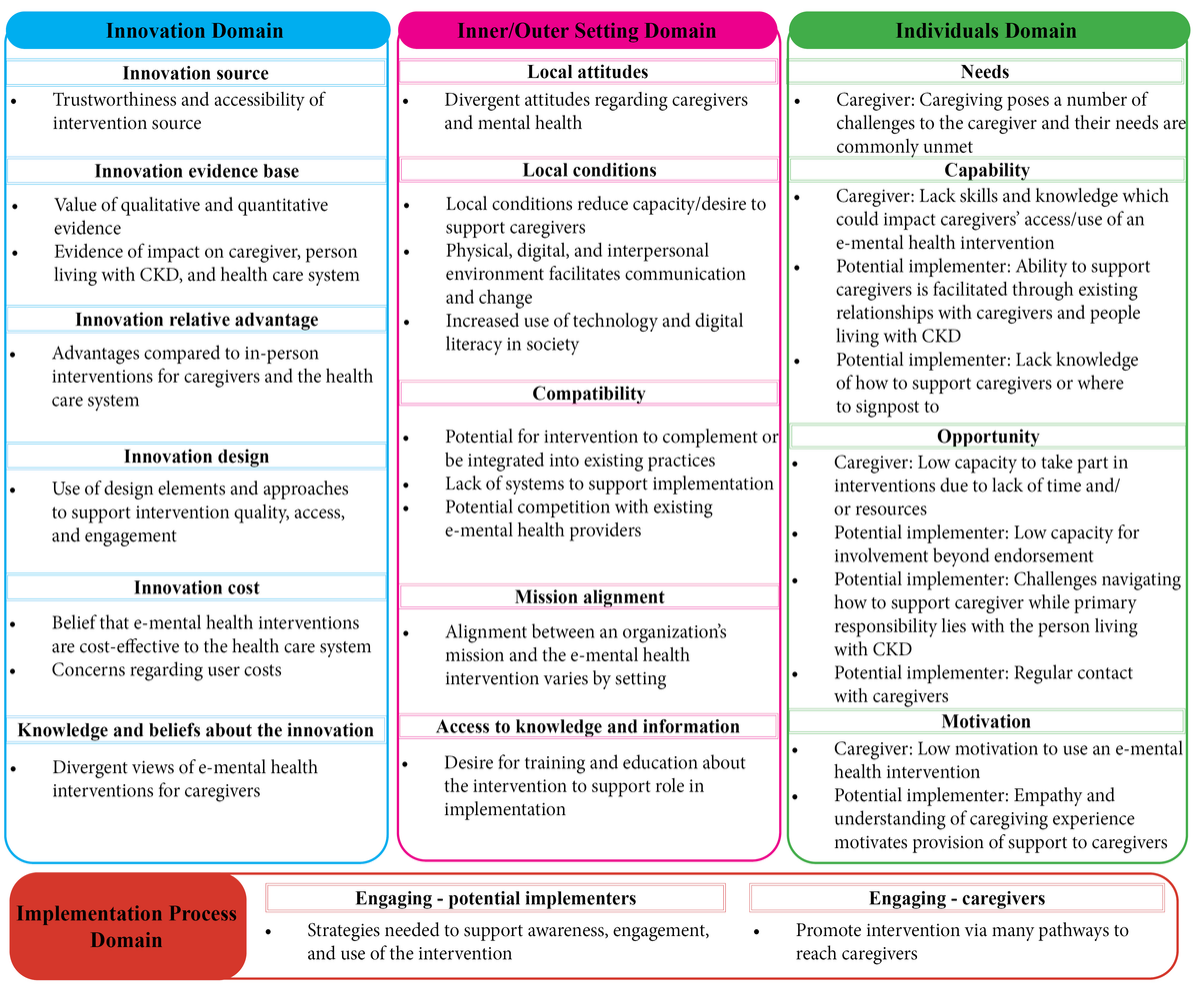
Consolidated Framework for Implementation Research (CFIR) domains and constructs with generic categories regarding implementation and design of e–mental health interventions for caregivers of people living with chronic kidney disease (CKD). The CFIR construct “Knowledge and beliefs about the innovation” is based on a construct from a previous version of the CFIR [30].

The analysis identified 29 generic categories related to 17 CFIR constructs (Figure 1 [30]). The coding tree with CFIR constructs, generic categories, and subcategories is presented in Multimedia Appendix 5.

Overall, potential implementers expressed similar perspectives regardless of their professional role. However, supporting quotations are provided alongside the potential implementers’ professional roles to help locate potentially important patterns in the data and facilitate interpretation. No disconfirming cases were identified. However, divergent views were expressed and reported where relevant. Additional supporting quotations to improve transparency are presented in Multimedia Appendix 5.

### CFIR Domain: Innovation

The *innovation* domain defines intervention characteristics to be considered when developing an e–mental health intervention optimized for implementation [32]. Data related to 6 CFIR constructs in the *innovation* domain were identified: innovation source, innovation evidence base, innovation relative advantage, innovation design, innovation cost, and knowledge and beliefs about the innovation.

#### Innovation Source

The trustworthiness of the innovation source (ie, the organization that visibly sponsors or implements the e–mental health intervention) was viewed as important to instill confidence in the intervention. Both nonprofit organizations and the NHS were considered trustworthy potential innovation sources. However, the patient focus of the NHS was raised as a potential barrier to caregivers accessing the intervention, given the current lack of support systems for caregivers. This indicates that nonprofit organizations may be better equipped to prioritize caregivers:

[...] it’d be lovely to think it was in the NHS. But that’s not always the right place to be. So sometimes the charities are better [...]. They can often publicize things more, if you knew where to signpost it, yes, I think that [charities] would probably be the best place. And also just, the carers [are] not the patient in the NHS, so how would they access it?

P3—kidney HCP

Private companies were viewed negatively as potential innovation sources, given the potential for the prioritization of profits over positive caregiver outcomes:

Well, I think if it was delivered by a private company, some people would treat it with a degree of skepticism. Because [there would] always be the fear, there’s a profit motive lying behind this or maybe it’s this, faceless uncaring company, that doesn’t really have carer’s interests at heart. Of course, I imagine most people probably would be fine with it. But I think you would find that there’s a number of people that maybe would be slightly more reluctant to do it if they thought there was a private company behind it.

P16—professional at a nonprofit organization for caregivers

#### Innovation Evidence Base

Potential implementers expressed the importance of establishing a research evidence base regarding the clinical effectiveness, acceptability, and cost-effectiveness of the intervention. Additional outcomes raised included process outcomes (eg, the number of users) and evidence that the intervention did not cause harm. Although professional groups expressed similar perspectives concerning the need to establish an evidence base, kidney HCPs also considered evidence of secondary benefits for the person living with CKD as desirable. Among mental HCPs, the existing evidence base for mental health interventions based on CBT was viewed as potentially minimizing the need for evidence related to the specific e–mental health intervention for CKD caregivers. Potential implementers valued both quantitative and qualitative evidence; however, they anticipated professionals from differing professional backgrounds may have stronger preferences for specific types of evidence:

My colleagues who are from the medical quantitative world would want very clear, very simple quantitative trial evidence and [evidence that] it was effective, I think. Otherwise they are not massively convinced by interventions, which is a shame, but yes.

P1—kidney HCP

#### Innovation Relative Advantage

e–Mental health interventions were viewed as having several advantages compared with in-person interventions, which could benefit both caregivers and the health care system. e–Mental health interventions were perceived as providing caregivers with flexible access to the intervention, which could help caregivers balance using the intervention with caregiving responsibilities, potentially minimizing their experiences of guilt. In addition, caregivers could access the intervention without leaving the house, which was perceived as beneficial for caregivers who may still be minimizing social contacts in response to the COVID-19 pandemic. e–Mental health interventions were perceived as providing a private and autonomous way for caregivers to access support without disclosing to HCPs or the person they care for that they need mental health support:

If it’s [the intervention’s] eas[ily] accessible and then more people would take it up, far more than would [be] phoning me to say “I’m struggling, can you help me?”. Some people like to keep a bit of a distance and not show, as they see [it], weakness that they’re not coping.

P2—kidney HCP

e–Mental health interventions were viewed as requiring fewer health care resources compared with in-person interventions, as the e–mental health intervention could operate with minimal staff support (eg, if the intervention was self-administered). In addition, the e–mental health intervention could provide immediate support to caregivers without waiting lists, given that the intervention could be less reliant on staff:

You don’t have to wait six weeks or more to actually get accepted. You could go on [to the intervention] and have a look and see if you can help yourself there [...] as opposed to waiting that long period. Because at the end of the day when somebody’s well-being is causing them concern, they don’t want to be told they are going to have [to] wait six to eight weeks before they can speak to somebody, they want to speak to somebody now.

P4—kidney HCP

#### Innovation Design

Several design elements and approaches were identified that could be applied to the design of the e–mental health intervention to ensure the intervention is of high quality and to enhance intervention access and engagement. The value of designing the intervention collaboratively with caregivers, implementers, and other professionals (eg, content experts) to enhance the quality and validity of intervention content was emphasized. Potential implementers also expressed the importance of designing an intervention that is easy to use and understand for people with different learning needs, providing extra support or a nondigital intervention version to people with lower digital literacy to increase accessibility. The e–mental health intervention was viewed as needing to incorporate strong safeguarding protocols to ensure caregivers in need of a higher level of support are referred to appropriate alternative interventions:

Because people come across, you know, just a bit low and then when you probe them it’s [their mental health difficulties] much more than you think. And that’s when you need to know that the system’s robust enough to pick up and, or we say this [intervention] isn’t appropriate, we need you to go back to someone.

P3—kidney HCP

The provision of additional support within the intervention was perceived as a way of enhancing users’ comprehension of intervention content, as a way of building trust, and as a strategy to support engagement. The types of support mentioned included support from a trained professional or automated messages. Support from a trained professional was viewed as a way to enhance engagement (eg, regular progress check-ins) and personalize the intervention (eg, provide personalized feedback to a user).

Potential implementers also expressed the importance of designing an intervention tailored to caregivers’ needs and contexts to ensure relevancy. For example, tailoring content to the context of caring for someone with CKD, caregiver’s location, preferences (optional peer support and dyadic activities), and background (eg, language, ethnicity, and gender).

#### Innovation Cost

The e–mental health intervention was viewed as having the potential to represent a cost-effective solution. Potential ways the e–mental health intervention could result in cost savings to the health care system included greater availability of informal care if caregivers’ well-being is supported and reduced time spent by health care staff responding to caregivers’ questions if the intervention contained renal-specific information:

I think that for people with milder presentations, I think it could be a really, I’m sure, cost-effective but really efficient way to deliver the kind of lower intensity interventions.

P10—mental HCP

However, concerns were expressed if the e–mental health intervention would only be available at a cost to the caregiver, given that potential implementers did not typically refer people to interventions with a cost and were aware many caregivers experience financial difficulties:

But, you know, as an NHS service it’s hard for us to promote things that then cost the patient or the relative money. You feel that you’re asking them to spend money [...] There would be a barrier certainly to people promoting it because again you probably would end up promoting it to people that you know can afford it. And a lot of our patients don’t have much money.

P7—kidney HCP

#### Knowledge and Beliefs About the Innovation

Potential implementers held divergent views and beliefs regarding the e–mental health intervention. e–Mental health interventions were perceived as benefiting caregivers in relation to improving caregivers’ well-being (eg, encouraging self-care and reducing isolation) and increasing caregivers’ knowledge. An e–mental health intervention specifically for caregivers was also viewed as validating the importance of the caregiving role and acknowledging the mental health impact the provision of informal care can cause:

It means that on one level it’s actually just quite useful to have an intervention about mental health that is specifically for carers. Because they [caregivers] can see that there’s something there tailored to them. The fact that an intervention has been created sort of legitimises and reinforces the importance of it. Because the carer might see, oh there’s a new app for carer mental health and it might make them reflect, maybe to a greater extent on their mental health. It sort of shows that it’s an important thing and someone has invested some time and money into integrating.

P16—professional at a nonprofit organization for caregivers

However, potential implementers also held negative views and beliefs about the e–mental health intervention. An e–mental health intervention was viewed as potentially not meeting the needs or preferences of all caregivers, for example, those with more severe mental health problems or who prefer in-person support. Given that not all caregivers may want or be able to use an e–mental health intervention, the importance of e–mental health interventions being offered as a choice with alternative interventions available was stressed:

For those it doesn’t work [for], what are you going to have in its place? And that would be my biggest concern.

P15—professional at a nonprofit organization for caregivers

Some potential implementers had past experiences with e–mental health, which made them perceive these interventions as impersonal and negatively impacting the therapeutic relationship.

### CFIR Domain: Inner/Outer Setting

The *inner/outer setting* domain describes the structural, cultural, and political context both within and outside of organizations that could influence implementation [32]. Data related to 5 CFIR constructs in the *inner/outer setting* domains were identified: local attitudes, local conditions, compatibility, mission alignment, and access to knowledge and information.

#### Local Attitudes

Potential implementers reported the presence of divergent views and attitudes regarding caregivers and their mental health. The value of caregivers was acknowledged, both in terms of caregivers’ role in supporting people living with CKD and in the relationship between caregiver well-being and the well-being of the person living with CKD:

Because there’s evidence suggesting that if a carer is struggling with their mental health, it’s going to have an impact on the physical health of the person that they’re looking after, and quite significantly depending on what’s wrong with the person.

P11—mental HCP

However, kidney HCPs noted that some of their colleagues view support for caregivers and the consideration of mental health needs as outside of their responsibility:

There is obviously a limit to the responsibility of a doctor and I think a lot of people feel it ends with the patient and doesn’t go beyond that.

P1—kidney HCP

In addition, potential implementers felt societal stigma surrounding mental health was decreasing and discussions about mental health were becoming normalized. This could help facilitate conversations between potential implementers and caregivers about the e–mental health intervention and support the uptake of the intervention among caregivers. However, it was also acknowledged that although stigma is decreasing, it is still present.

#### Local Conditions

Potential implementers emphasized that local conditions could reduce the capacity and desire to support caregivers. Despite acknowledgment of the importance of caregivers, providing support to caregivers was often a low priority in society, with few dedicated services available. Poor funding for support services was also raised as a barrier, given that the available caregiver support changes regularly, making it difficult to refer caregivers to services. Capacity constraints within the health care system (eg, loss of staff to provide mental health support and long waitlists for support) and environments lacking a desire for change, coupled with persisting impacts from the COVID-19 pandemic, were perceived as barriers to implementation:

And I think we’re just very much firefighting. We have way too many patients we’re understaffed for. So then, do you know, to- We don’t feel like we meet the needs of what we should be doing for our patients, let alone their caregivers.

P7—kidney HCP

Despite capacity constraints, the physical (ie, shared office space), digital (ie, WhatsApp (Meta Platforms, Inc), email, and shared databases with resources), and interpersonal (ie, relationships with colleagues) environments were perceived as facilitating communication among potential implementers both within and across settings. This could create a supportive environment for change and information dissemination. In addition, increased technology use and societal digital literacy levels were viewed as supporting e–mental health implementation:

I work closely with the transplant specialist nurse [...] and she’s always a really, really good sounding block if I ever say, “oh you know, I’ve got a patient I’m concerned about it”, she will often say, “Tell me what your issue is”. We’ll talk it through and then she always suggests things if I haven’t already come up with them. She’s got a wealth of knowledge and she’s a really good person to go to. But also we’ve [got] supportive care nurses. There are lots of people and I work really closely with all the different sort of teams of people.

P5—kidney HCP

#### Compatibility

The e–mental health intervention was viewed as having the potential to be integrated into existing practices and workflows. Kidney and mental HCPs felt that the e–mental health intervention could fit well within some health care delivery models (ie, stepped care and transplant psychosocial assessment). Potential implementers were already engaged with caregiver referral, and the e–mental health intervention was viewed as a resource to enhance this practice. In health care settings, there was often no system to record if caregivers requested, needed, or had been referred to support but kidney HCPs suggested caregiver support could be integrated into electronic medical records (eg, a tick box to indicate if a caregiver was referred to support and a reminder for HCPs to inform the caregiver about available support):

It [the intervention] could be easily fitted in without taking any more time. I think if anything it would make things, it would speed things up because you’d have, instantly know what to say, how to signpost them correctly without it just relying on that health care professional’s knowledge and confidence, you know, that it’s done correctly really.

P6—kidney HCP

Potential implementers working in settings providing services to broader populations (eg, caregivers of people living with any chronic or mental health condition or adults with mental health problems) were unsure how many of their existing clients would be suitable for the e–mental health intervention, and systems were not in place to identify people specifically caring for someone with CKD:

And the other thing that I wouldn’t be sure of is how [many] people [caregivers of people with CKD] we’ve got. [...] when anybody registers with us, we [don’t] ask them “why are you caring for this individual?”

P15—professional at nonprofit for caregivers

Competition between this e–mental health intervention and existing e–mental health providers was mentioned as a potential barrier to implementation by mental HCPs and professionals at nonprofit organizations. However, the only setting with an identified existing e–mental health provider was NHS Talking Therapies.

#### Mission Alignment

The alignment between an organization’s mission and the e–mental health intervention varied by setting. The only setting where it was explicitly mentioned that an e–mental health intervention for caregivers could align with the organization’s mission was at nonprofit organizations supporting caregivers:

Yes so it’s set out in our aim really. You know [...] we understand that the kidney journey for a patient isn’t just the patient. It’s a whole family. [...] So all of our services support the caregiver as well as the patient.

P17—professional at a kidney-specific nonprofit organization

Potential implementers working within NHS Talking Therapies recognized that although caregivers were not a specific target population, the provision of e–mental health interventions for caregivers could align with their mission of increasing the uptake of mental health services. However, it was also acknowledged that caregivers were not currently considered a priority group.

#### Access to Knowledge and Information

Potential implementers expressed a desire to have access to training and information about the e–mental health intervention. They wanted to understand the intervention’s purpose and content and to access the intervention themselves. Building familiarity with the intervention was perceived as positively influencing beliefs regarding intervention quality and its ability to benefit caregivers. The availability of physical materials (eg, flyers) and a point of contact with someone having more extensive knowledge of the intervention were perceived as facilitating implementation and endorsement of the intervention:

Have a contact that everyone knows about who’s a good go to person, who is a bit more knowledgeable on it. Have a good backup within the e-provider for if there were queries about how something worked or things that went wrong, in terms of IT.

P10—mental HCP

### CFIR Domain: Individuals

The *individuals* domain refers to the characteristics and qualities of caregivers and potential implementers that could influence their ability to use or implement the e–mental health intervention [32]. Data related to 4 CFIR constructs in the *individuals* domain were identified: needs, capability, opportunity, and motivation.

#### Needs

Caregiving was viewed as a challenging experience, impacting caregivers’ physical and mental health, thus supporting the need for the intervention. However, caregivers were perceived as focusing so much on their caregiving responsibilities that they may neglect their own well-being and feel reluctant to seek support from HCPs (eg, feel they should be able to cope, perceive HCPs as having limited time). Therefore, caregivers were viewed as often having unmet support needs:

We pick up from carers, that many carers feel guilty actually, guilty that they’re not providing enough care or good enough care. They’re so focused on the needs of the person that they look after. I think many carers will actually neglect their own mental health.

P16—professional at a nonprofit organization for caregivers

#### Capability

Potential implementers perceived that caregivers may lack the skills and knowledge needed to access or use an e–mental health intervention. This is primarily related to concerns regarding digital literacy, which was viewed as being closely related to caregivers’ age (eg, assuming older caregivers have lower digital literacy). In addition, caregivers were perceived as not always being aware that they were in a caregiving role; therefore, they may not access an intervention promoted for caregivers. Given the long trajectory of CKD, many kidney HCPs had longstanding relationships with caregivers and people living with CKD, which could facilitate the identification of caregivers in need of support and referral of caregivers to an e–mental health intervention:

One of the nice things about [...] looking after people with kidney diseases is that I get to know people. So it’s quite easy to build a relationship, where you can say, “and how are things for you” to a caregiver.

P1—kidney HCP

Currently, both kidney and mental HCPs feel they lack knowledge of where to refer caregivers for support.

#### Opportunity

Caregivers were anticipated to lack the capacity to engage with an e–mental health intervention because of the lack of time, energy, and resources (eg, no computer access and inability to afford the internet). Potential implementers often came into contact with caregivers as part of their role, which would provide them with the opportunity to refer caregivers to the intervention. However, they perceived themselves as lacking the capacity to be involved with implementation beyond the endorsement or referral of caregivers to the intervention owing to a lack of time and resources. Navigating the responsibility potential implementers have for the person living with CKD was recognized as a potential barrier to implementing or endorsing the intervention. Additional barriers raised included people with CKD blocking access to their caregivers:

It could cause issues if you’ve got a family member who wants to get some support for themselves when the patient’s thinking “why are you suffering when I’m the patient?” It could cause tension possibly.

P5—kidney HCP

#### Motivation

Caregivers’ motivation to use an e–mental health intervention was expected to be low, given that caregivers often prioritize other responsibilities over self-care and may hold negative views about mental health interventions (eg, caregivers may view accessing mental health support as a weakness). Empathy for caregivers stemming from personal experience working with caregivers or providing unpaid care to a family member or friend was a source of motivation to support caregivers:

I mean as well as our volunteers, most of them have come through the carer background route. A lot of the staff has as well. You know, at least half the staff here are currently carers or have been carers or are going to be carers really shortly, you know. It’s just the way it is. So not that I should say that gives us a, you know we understand everybody’s position, but it gives us a bit of an insight into what’s going on.

P15—professional at a nonprofit organization for caregivers

### CFIR Domain: Implementation Process

The *implementation process* domain describes activities and strategies that could be used to support the implementation and uptake of the e–mental health intervention [32]. Data related to 2 CFIR constructs in the *implementation process* domain were identified: engaging–potential implementers and engaging–caregivers.

#### Engaging: Potential Implementers

To engage potential implementers in intervention delivery or endorsement, potential implementers felt strategies would be needed to increase awareness of the intervention and encourage potential implementers to use and engage with the intervention. Potential strategies identified included having a fast and easy referral pathway, continuous efforts to raise awareness and remind potential implementers of the intervention, and ensuring all members of clinical multidisciplinary teams are aware of the intervention, given that many different HCPs come into contact with caregivers:

Just as long as you had a clear pathway, you know, with the right element of referral. If there’s a very simple referral form maybe, that’s, you know something like that, but very simple. Not complex because we have plenty of them. Just easy, make it easy. Please make it easy. That’s it.

P11—mental HCP

#### Engaging: Caregivers

Potential implementers felt engaging caregivers to use the intervention would be supported by promoting the intervention via multiple pathways (eg, advertisements, newsletters, and in-person communication) and in multiple settings (eg, health care settings, nonprofit organizations, and social media):

There’s like national patient magazines that we’d put it in and then posters at the dialysis units in the waiting rooms. And leaflets in waiting rooms [...]. And then carrying some with us so that when we’re seeing patients we can hand them out.

P7—kidney HCP

## Discussion

### Principal Findings

#### Overview

This study identified several implementation factors within all domains of the CFIR that require consideration during the design and implementation of an e–mental health intervention for caregivers of people living with CKD. Some identified factors align with existing caregiving literature that has similarly identified the relative advantage of e–mental health interventions (eg, flexible access), the barriers caregivers may experience if accessing an e–mental health intervention (eg, low digital literacy and low motivation), the presence of both positive (eg, beneficial for caregivers) and negative (eg, impersonal) views of e–mental health interventions, and the importance of designing e–mental health interventions that are easy to use and contain tailored content [10,39,40].

Key implementation factors related to CFIR constructs, which have been less frequently explored in the existing literature, were also identified. In relation to the CFIR construct innovation evidence base, the need to obtain qualitative and quantitative evidence regarding the e–mental health intervention to meet different preferences among potential implementers can be used to guide data collection decisions in future research regarding the effectiveness and acceptability of the developed intervention. The involvement of potential implementers throughout all phases of intervention development and evaluation is recommended by the MRC framework [11] and could be a way to ensure that data relevant to potential implementers is collected. Within the *individuals* domain, although the characteristics of caregivers that influence their ability to use an e–mental health intervention have often been explored in the literature [10,39], the characteristics of potential implementers are seldom reported. Among potential implementers, characteristics including lack of knowledge on how to support caregivers and challenges offering caregivers support while remaining focused on the person living with CKD were identified as potential barriers to implementation. In addition, several potential implementation barriers (eg, low priority of services for caregivers) and facilitators (eg, work environments that support communication) related to the implementation context were identified. This addresses an important gap in the literature regarding the implementation of e–mental health interventions for caregivers [10]. Addressing the implementation factors identified in this study by identifying strategies to overcome barriers and leverage facilitators should be considered as intervention development and implementation planning continue.

#### Fit Between the Intervention and Implementation Context

Within the *inner/outer setting* domain of the CFIR, potential implementers’ views illustrated the potential for the e–mental health intervention to fit within local attitudes and conditions. For example, how the purpose and format of the intervention aligned with positive views of caregivers and the increased use of technology in society. Potential implementers also identified the potential compatibility between the intervention and existing health care delivery models and work routines. Integration between interventions and existing systems and care pathways has been identified as an implementation determinant for other e–mental health interventions for caregivers [41,42]. Therefore, efforts should be made when developing the e–mental health intervention to further consider how to integrate the intervention with existing systems in place within the implementation setting.

Spanning the *inner/outer setting*, *individuals*, and *implementation process* domains of the CFIR, potential implementers referred to relationships with both caregivers and colleagues as potential implementation facilitators. HCPs had longstanding relationships with caregivers, which were identified as potentially facilitating conversations about the caregivers’ well-being and the e–mental health intervention. Potential implementers had relationships with professionals within and outside of their workplaces, facilitating information sharing and collaboration. These existing relationships could be used to support the uptake of the e–mental health intervention through the dissemination of information, especially if professionals with greater influence over their peers (ie, opinion leaders) were engaged during implementation [43]. Importantly, consideration of social networks during intervention dissemination and implementation is increasingly being explored within implementation research as a way to influence implementation outcomes (eg, acceptability and adoption) and inform the design, implementation, dissemination, and sustainability of interventions [44,45]. The findings suggest that the social networks of potential implementers should be further explored once an implementation setting has been identified to gain insights regarding who to strategically involve during implementation to facilitate the spread and uptake of the intervention [46].

#### Need for Capacity Building

Within the *inner/outer setting* and *individuals* domains of the CFIR, several anticipated barriers to future implementation were related to the lack of system- and individual-level capacity to implement the e–mental health intervention. Potential implementation settings, especially health care settings, were identified as lacking formal systems and protocols related to referral and provision of caregiver support. Creating formal systems that support caregivers could help build system-level capacity to support caregivers by creating efficient pathways to refer caregivers to existing services and lead to more consistent integration of caregiver support into HCPs’ practice [47,48]. Formal systems related to caregiver support may be especially relevant, given that HCPs can have different views regarding their roles and responsibilities related to caregivers. Systematic identification of caregivers is a common challenge across many settings because of barriers such as lack of time and skills to support caregivers among HCPs, caregivers not identifying as being in a caregiving role, and the absence of systems to document caregiver needs [47-49]. Both Carers UK and the NHS recommend the development of a systematic approach within the health care system to identify and support caregivers [50,51]. This reinforces the need for system-level change to create a context with a greater capacity to support caregivers.

Given that potential implementers expressed having limited capacity to implement or endorse an e–mental health intervention, identifying strategies to build an individual-level capacity to endorse the intervention will be a key consideration when developing future implementation strategies. For example, although kidney HCPs recognize the value of caregivers, supporting caregivers could conflict with their responsibility to the person with CKD. Findings suggested that providing evidence regarding the importance of caregivers’ mental health in relation to outcomes for people with CKD could motivate more kidney HCPs to incorporate caregiver support into their practice. Although there is evidence that caregiver interventions can benefit care recipients, care recipient outcomes are not always incorporated into their evaluation [52]. As such, the findings suggest that future research evaluating the effectiveness the e–mental health intervention should also measure the indirect impact of the intervention on the care recipient, which may act as a facilitator for future implementation. In addition, the provision of materials and training to enhance knowledge of the intervention and how to communicate with caregivers could support implementation. Providing education about new interventions and building implementers’ self-efficacy are strategies that have been shown to be important when implementing e–mental health interventions for other caregiving populations [53]. Future work focused on developing interventions and identifying implementation strategies could benefit from theories and tools such as the behavior change wheel [31] and Behaviour Change Intervention Ontology [54]. The Expert Recommendations for Implementing Change [55] may also be used to guide the selection of implementation strategies to build system- and individual-level capacity to implement a future e–mental health intervention.

#### Applying an Equity Lens to Intervention Design

Within the *Innovation* domain of the CFIR, potential implementers expressed concerns about how an e–mental health intervention could be designed to better meet the different needs and skill levels of potential users to ensure the intervention is accessible and does not exclude caregivers from accessing the support it provides. The application of an equity lens to intervention design and implementation could be adopted to ensure equity remains in focus throughout intervention development. The PROGRESS framework outlines 8 factors to consider when applying an equity lens to designing and implementing interventions, namely place of residence, race, ethnicity, culture and language, occupation, gender, religion, education, socioeconomic status, and social capital [56]. Potential implementers have already identified key considerations related to the socioeconomic status and education factors of the PROGRESS framework. To address potential access barriers when designing the intervention, it may be relevant to engage existing organizations to have pathways in place to support caregivers in obtaining the equipment (eg, IT equipment loan programs) and digital skills training needed to use the e–mental health intervention [57]. The CFIR does not explicitly have an equity focus, although factors related to equity can be captured within the framework [32]. To enhance the equity focus as implementation continues to be explored, health equity domains could be incorporated into the CFIR, as has been done with other implementation frameworks [58].

### Limitations

Many different professionals (eg, kidney HCPs, mental HCPs, and staff at nonprofit organizations) would be involved in the implementation of an e–mental health intervention. Therefore, we sought to include professionals working in different roles in various potential implementation settings. Although we achieved diversity in relation to potential implementers’ professional backgrounds and workplaces, this also created heterogeneity, which could have resulted in highly divergent views on the e–mental health intervention. However, given that this study explored the hypothetical implementation of an e–mental health intervention, a heterogeneous sample provides evidence that may be applicable to several different potential implementation settings.

The views of potential implementers were based on a brief intervention description, which could limit the ability of potential implementers to provide more specific feedback. However, this study was intended to be exploratory, and potential implementers will continue to be engaged throughout the intervention development process. Finally, primarily using a deductive coding approach can promote data being forced into the framework and discourage the identification of categories that do not fit within the framework. However, adopting a primarily deductive and descriptive approach aligns with our pragmatic objective of describing factors that should be considered when designing and implementing an e–mental health intervention for caregivers. In addition, deductive coding using the CFIR ensured a systematic consideration of implementation determinants identified in the wider implementation literature [30] during data analysis.

### Conclusions

This study provides an example of an approach to begin exploring factors influencing implementation from a very early stage of intervention development. It has identified several factors that could influence the implementation of e–mental health interventions for caregivers that are seldom explored in the literature (eg, local attitudes and local conditions). The findings will be used to inform the development of an e–mental health intervention for caregivers of people living with CKD and anticipated implementation barriers and facilitators could inform the selection of implementation strategies to optimize successful implementation [55]. Digital intervention development frameworks, such as the Integrate, Design, Assess, and Share framework [59], should also be considered as e–mental health intervention development continues.

## Appendix

Multimedia Appendix 1 Standards for Reporting Qualitative Research.

Multimedia Appendix 2 Interview guide.

Multimedia Appendix 3 Potential implementer background characteristics.

Multimedia Appendix 4 Construct definitions.

Multimedia Appendix 5 Categories and quotes.

## Abbreviations

CBT: cognitive behavioral therapy
CFIR: Consolidated Framework for Implementation Research
CKD: chronic kidney disease
HCP: health care professional
MRC: Medical Research Council
NHS: National Health Service
NPO: nonprofit organization
