# Supplementary material for: Potential Implementers’ Perspectives on the Development and Implementation of an e–Mental Health Intervention for Caregivers of Adults With Chronic Kidney Disease: Qualitative Interview Study
Source: JMIR Hum Factors. 2023 Nov 17;10:e51461. doi: 10.2196/51461 (PMC10692875; doi:10.2196/51461)
Supplement: Multimedia Appendix 2 [file humanfactors_v10i1e51461_app2.pdf]

Multimedia Appendix 2: Intervention description and interview guide

- Multimedia Appendix 2a: Intervention description provided to participants
- Multimedia Appendix 2b: Interview guide

Multimedia Appendix 2a: Intervention description provided to participants

Below is a brief description of how an e-mental health intervention for informal caregivers of people with chronic kidney conditions could be delivered and implemented.

We would like to develop an e-mental health intervention specifically designed for informal caregivers of people with chronic kidney conditions. We would like to hear your views on what you think might work best e.g., what type of support informal caregivers of people with chronic kidney conditions need, how the support might be provided, who could provide/deliver the e-mental health intervention, and what barriers to implementing such an intervention might exist.

<u>What might the e-mental health intervention look like?</u>

The intervention will be designed to help informal caregivers of people with chronic kidney conditions with their psychological wellbeing, for example feeling low, down, anxious, worried, and stressed.

The intervention will be based on evidence based techniques from cognitive behavioural therapy.

The intervention will also include other important information that caregivers might find helpful, for example, returning to work, diet and exercise, sexual relations and intimacy, and information about relevant support services.

The intervention will be designed to last for about 6 weeks and will include weekly homework exercises.

The intervention will be delivered electronically. For example, accessed via a website or a smart phone application. The intervention can consist of text, illustrations, film, and audio files. Typically e-mental health interventions include different modules, for example, with specific techniques from cognitive behavioural therapy, and information that might be helpful for informal caregivers of people with chronic kidney conditions.

The informal caregivers will be supported by someone trained in how to provide support and guidance to people using e-mental health interventions. Support will be received on a regular basis (for example, weekly) and can be provided face-to-face, over the telephone, via video-conference, or via email.

The person providing support and guidance will be trained. However, they are not required to have a core physical health or mental health professional qualification.

The person providing support and guidance may be involved in activities such as:

- Assessing informal caregivers to understand what psychological support they need
- Support/guide informal caregivers to use the cognitive behavioural therapy based techniques within the e-mental health intervention
- Signpost informal caregivers to other sources of support (including health and social care professionals and other sources of community based support)

If support and guidance is provided face-to-face, this could be in a variety of different locations, for example, in a renal/satellite unit, at the hospital, at a GP practice, within a mental health service, or within a community setting.

Multimedia Appendix 2b: Interview guide

1) Please can you tell me a little bit about your role within [insert organisation name]?
   a) PROMPTS: experience working with people who have a chronic kidney disease, experience working with informal caregivers
2) To what extent is psychological support for informal caregivers something that your [insert organisation name] supports?
   a) What gets in the way of psychological support being something that your [insert organisation name] supports?
   b) What facilitates psychological support being something that your [insert organisation name] supports?
3) In what ways do you think an e-mental health intervention, as described in the information we have provided you with, could meet the needs of informal caregivers?
4) What barriers do you think informal caregivers might experience to participate in an e-mental health intervention? What might help overcome these barriers?
5) What impact do you think an e-mental health intervention can have on informal caregivers of people with chronic kidney disease?
6) How might an e-mental health intervention fit with the work that [insert organisation name] does? (e.g., policies, guidelines, existing programmes/interventions, workforce?)
7) In what ways would an e-mental health intervention differ from the support currently made available to informal caregivers?
8) Are you aware of any other similar programmes that exist to provide psychological support for informal caregivers? If yes:
   a) How are these programmes delivered (e.g., by whom, how is it delivered, opinions on the existing programme)?
   b) What advantages does the proposed e-mental health intervention have compared to existing programmes?
   c) What disadvantages does the proposed e-mental health intervention have compared to existing programmes?

9) What roles can you envisage [insert organisation/professional role name] having with the proposed e-mental health intervention. For example, can you envisage **endorsing the intervention,** for example, referring, informing, advertising, and promoting the intervention, or can you imagine **implementing** the intervention, for example, providing access to the intervention and supporting intervention delivery?

If endorsing the intervention:

   a) In what ways might [insert organisation/professional role name] endorse the intervention?
      i) What might stop [insert organisation/professional role name] from endorsing the intervention?
      ii) What might help [insert organisation/professional role name] endorse the intervention?
   b) What kind of supporting evidence or proof is needed about the effectiveness of the intervention to get staff on board?
   c) How would you describe the culture (general beliefs, values, assumptions that people embrace) of [insert organisation name]?
      i) Do you feel like the culture of your own unit/department/group is different from the overall organisation? In what ways?
      ii) How do you think the culture (general beliefs, values, assumptions that people embrace) of [insert organisation name] will affect your organisation being able to endorse the intervention? Can you describe an example that highlights this?

(1) To what extent are new ideas embraced and used to make improvements in [insert organisation name]? Can you describe a recent example?

If implementing the intervention:

d) How might [insert organisation/professional role name] be involved in implementing the intervention? For example:
   i) Providing support and guidance to informal caregivers?
   ii) Who should provide this support and guidance?
   iii) Where would this support and guidance be provided?
e) How would you describe the culture (general beliefs, values, assumptions that people embrace) of [insert organisation name]?
   i) Do you feel like the culture of your own unit/department/group is different from the overall organisation? In what ways?
   ii) How do you think your organisation's culture (general beliefs, values, assumptions that people embrace) will affect the implementation of the intervention? Can you describe an example that highlights the culture of your organisation?
   iii) How would implementing the e-mental health intervention effect current working practices within [insert organisation name]
f) How could delivering the e-mental health intervention be integrated into your current working practices?
   i) Who within [insert organisation name] would be important to involve in implementing the intervention?
   ii) What do you think influential stakeholders in your own unit/department/group would think of the intervention?
   iii) What support would be needed from leaders in [insert organisation name] to make implementation successful?
g) What kind of supporting evidence or proof is needed about the effectiveness of the intervention to get staff on board?
h) What kind of support would be required to help people in [insert organisation name] understand what the intervention is and how it should be used?
i) Do you expect to have sufficient resources to implement and administer the intervention?
j) What do you think might be some of the main barriers to implementing such an intervention in your workplace (e.g., funding, training, decision making, external policies, and incentives)
k) What do you think might help overcome barriers to implement such an intervention?

10) Is there anything else you would like to add that have not been addressed about the proposed e-mental health intervention that you think is important for me to know?
