# Supplementary material for: Potential Implementers’ Perspectives on the Development and Implementation of an e–Mental Health Intervention for Caregivers of Adults With Chronic Kidney Disease: Qualitative Interview Study
Source: JMIR Hum Factors. 2023 Nov 17;10:e51461. doi: 10.2196/51461 (PMC10692875; doi:10.2196/51461)
Supplement: Multimedia Appendix 3 [file humanfactors_v10i1e51461_app3.pdf]

Multimedia Appendix 3: Background characteristics of potential implementers (n = 18)

ID number	Age (years)	Gender	Role	Time in current role (years)	Experience working with caregivers of people with CKD	Experience working with people with CKD	Experience working with people with mental health problems	Experience working with caregivers of people with other chronic diseases
1	40-49	F	Kidney HCP	0-5	Yes	Yes	Yes	No
2	40-49	M	Kidney HCP	6-10	Yes	Yes	Yes	Yes
3	50-59	F	Kidney HCP	11-15	Yes	Yes	Yes	No
4	50-59	F	Kidney HCP	0-5	Yes	Yes	Yes	Yes
5	50-59	F	Kidney HCP	6-10	Yes	Yes	Yes	No
6	30-39	F	Kidney HCP	6-10	Yes	Yes	Yes	Yes
7	30-39	F	Kidney HCP	0-5	Yes	Yes	Yes	No
8	50-59	F	Kidney HCP	11-15	Yes	Yes	Yes	Yes
9	50-59	F	Kidney specific mental HCP	11-15	Yes	Yes	Yes	Yes
10	50-59	F	Mental HCP	0-5	No	Yes	Yes	Yes
11	40-49	F	Mental HCP	0-5	No	No	Yes	Yes

12	50-59	F	Mental HCP	6-10	Yes	No	Yes	Yes
13	50-59	F	NPO professional-caregiver	6-10	Yes	Yes	Yes	Yes
14	60-65	F	NPO professional - caregiver	11-15	Yes	Yes	Yes	Yes
15	50-59	M	NPO professional - caregiver	0-5	Yes	Yes	Yes	Yes
16	20-29	M	NPO professional - caregiver	0-5	No	No	No	Yes
17	50-59	F	NPO professional - kidney	6-10	Yes	Yes	Yes	No
18	50-59	M	NPO professional - kidney	0-5	Yes	Yes	Yes	Yes

Note. Ages and time in current role provided in bandings to protect confidentiality. Abbreviations: CKD: chronic kidney disease; F: female; M: male; HCP: healthcare professional; NPO: non-profit organisation
