# Supplementary material for: Potential Implementers’ Perspectives on the Development and Implementation of an e–Mental Health Intervention for Caregivers of Adults With Chronic Kidney Disease: Qualitative Interview Study
Source: JMIR Hum Factors. 2023 Nov 17;10:e51461. doi: 10.2196/51461 (PMC10692875; doi:10.2196/51461)
Supplement: Multimedia Appendix 4 [file humanfactors_v10i1e51461_app4.pdf]

Multimedia Appendix 4: Coding constructs from the CFIR with general and tailored definitions

CFIR domain	CFIR construct	General definition[a]	Tailored definition
Innovation domain	Innovation source	The degree to which the group that developed and/or visibly sponsored use of the innovation is reputable, credible, and/or trustable.	The degree to which group(s) that could potentially develop, implement, and/or visibly sponsor use of the e-mental health intervention are reputable, credible, and/or trustable.
	Innovation evidence-base	The degree to which the innovation has robust evidence supporting its effectiveness.	The evidence potential implementers want/need that supports effectiveness of the e-mental health intervention.
	Innovation relative advantage	The degree to which the innovation is better than other available innovations or current practice.	The degree to which the e-mental health intervention could be better than other innovations (e.g. in-person interventions) or current practice.
	Innovation design	The degree to which the innovation is well designed and packaged, including how it is assembled, bundled, and presented.	How the e-mental health intervention should be designed and packaged, including how it is assembled, bundled, and presented.
	Innovation cost	The degree to which the innovation purchase and operating costs are affordable.	Perceptions regarding the cost of the e-mental health intervention.
	Knowledge and beliefs about the innovation[b]	Individuals' attitudes toward and value placed on the innovation, as well as familiarity with facts, truths, and principles related to the innovation.	Potential implementers' attitudes toward and value placed on the e-mental health intervention, as well as perceived knowledge, truths, principles related to the e-mental health intervention.
Inner/Outer setting domain	Local attitudes	The degree to which sociocultural values (e.g., shared responsibility in helping recipients) and beliefs (e.g., convictions about the worthiness of recipients) encourage the Outer Setting to support implementation and/or delivery of the innovation.	The degree to which sociocultural values (e.g., shared responsibility in helping caregivers, supporting mental health) and beliefs (e.g., convictions about the worthiness of caregivers) encourage or discourage the Outer/Inner setting to potentially support implementation, delivery, and/or sponsorship of the e-mental health intervention.
	Local conditions	The degree to which economic, environmental, political, and/or technological conditions enable the Outer Setting to support implementation and/or delivery of the innovation.	The degree to which economic, environmental, political, and/or technological conditions enable or inhibit the Outer/Inner setting to potentially support implementation, delivery, and/or sponsorship of the e-mental health intervention.

	Compatibility	The degree to which the innovation fits with workflows, systems, and processes.	The potential fit between the e-mental health intervention and existing workflows, systems, and processes.
	Mission alignment	The degree to which implementing and delivering the innovation is in line with the overarching commitment, purpose, or goals in the Inner Setting.	The degree to which implementing, delivering and sponsoring the e-mental health intervention could be in line with the overarching commitment, purpose, or goals in the Outer/Inner setting.
	Access to knowledge and information	The degree to which guidance and/or training is accessible to implement and deliver the innovation.	The characteristics of guidance and/or training that should be available to implement, delivery or sponsor the e-mental health intervention.
Individuals domain – Characteristics subdomain	Need	The degree to which the individual(s) has deficits related to survival, well-being, or personal fulfilment, which will be addressed by implementation and/or delivery of the innovation.	The degree to which the caregivers or potential implementers have deficits related to survival, well-being, or personal fulfilment, which could be addressed by implementation, delivery, and/or sponsorship of the e-mental health intervention.
	Capability	The degree to which the individual(s) has interpersonal competence, knowledge, and skills to fulfill Role.	The degree to which the caregiver or potential implementer has interpersonal competence, knowledge, and skills to participate in, implement, deliver, or sponsor the e-mental health intervention.
	Opportunity	The degree to which the individual(s) has availability, scope, and power to fulfill Role.	The degree to which the caregiver or potential implementer has availability, scope, power, and access to resources to participate in, implement, deliver, or sponsor the e-mental health intervention.
	Motivation	The degree to which the individual(s) is committed to fulfilling Role.	The degree to which the caregiver or potential implementer is committed to participate in, implement, deliver, or sponsor the e-mental health intervention.
Implementation process domain	Engaging - Potential implementers[c]	The degree to which individuals attract and encourage deliverers to serve on the implementation team and/or to deliver the innovation.	Strategies that could be used to attract and encourage potential implementers to serve on the implementation team, deliver, and/or sponsor the e-mental health intervention.

	Engaging - Innovation recipients	The degree to which individuals attract and encourage recipients to serve on the implementation team and/or participate in the innovation.	Strategies that could be used to attract and encourage caregivers to serve on the implementation team and/or participate in the e-mental health intervention.

[a]General definitions are the original definitions from the updated CFIR framework found in Damschroder, L.J., Reardon, C.M., Widerquist, M.A.O., Lowery, J., 2022. The updated Consolidated Framework for Implementation Research based on user feedback. Implement. Sci. 17, 75. https://doi.org/10.1186/S13012-022-01245-0.

[b]Knowledge and beliefs about the innovation was added as a construct based on a construct from the first version of the CFIR framework. The general definition reflects the definition from the original CFIR framework found in Damschroder, L.J., Aron, D.C., Keith, R.E., Kirsh, S.R., Alexander, J.A., Lowery, J.C., 2009. Fostering implementation of health services research findings into practice: a consolidated framework for advancing implementation science. Implement. Sci. 4, 50. https://doi.org/10.1186/1748-5908-4-50.

[c]Given the specific role of professionals would have during implementation of the e-mental health intervention was not established, the more generic role of potential implementers was created.
