# Supplementary material for: Potential Implementers’ Perspectives on the Development and Implementation of an e–Mental Health Intervention for Caregivers of Adults With Chronic Kidney Disease: Qualitative Interview Study
Source: JMIR Hum Factors. 2023 Nov 17;10:e51461. doi: 10.2196/51461 (PMC10692875; doi:10.2196/51461)
Supplement: Multimedia Appendix 5 [file humanfactors_v10i1e51461_app5.pdf]

Multimedia Appendix 5: Generic and sub-categories with additional supporting quotes

- Additional table 1: Generic, and sub-categories regarding implementation and design of e-mental health (e-MH) interventions for caregivers of people living with CKD within CFIR constructs

- Additional table 2: Additional supporting quotes from potential implementers for each CFIR construct

Additional table 1: Generic, and sub-categories regarding implementation and design of e-mental health interventions (e-MH) for caregivers of people living with CKD within CFIR constructs.

CFIR construct	Generic category	Sub-category
Innovation source	Trustworthiness and accessibility of intervention source	
Innovation evidence-base	Value of qualitative and quantitative evidence	● Qualitative (e.g. user and provider testimonials) and quantitative evidence valued<br>● Evidence preferences anticipated to vary among professionals (e.g. some may only value data from randomized controlled trials)
	Evidence of impact on caregiver, person living with CKD, and healthcare system	● Evidence of impact on objective (e.g. depression scale) and subjective (e.g. how much they liked the intervention) measures for caregivers<br>● Process outcomes that are fed back to implementers (e.g. number of users, number who complete the intervention)<br>● Evidence of intervention safety and that it does not cause harm<br>● Evidence of impact on person living with CKD<br>● Evidence of cost-effectiveness<br>● Existing evidence-base for internet-based CBT reduces the need for additional evidence
Innovation relative advantage	Advantages compared to in-person interventions for caregivers and the healthcare system	● Minimal healthcare resources required (e.g. less reliant on staff) which can facilitate efficient intervention access<br>● Flexible intervention access can better fit within caregiver's routines<br>● Sense of privacy and autonomous intervention access (i.e. self-referral) for caregivers
Innovation design	Use of design elements and approaches to support intervention quality, access, and engagement	● Provision of support to enhance understanding of intervention content, build trust, and support regular engagement with the intervention<br>● Collaboration to design the intervention with potential users and implementers to enhance intervention validity and quality<br>● Tailoring materials to needs and context (e.g. geographic region, caregiver's background (i.e. language, ethnicity, gender), CKD caregiving, individual content preferences)<br>● Provision of strong safeguarding and security protocols<br>● Provision of alternative intervention options to those who cannot or do not want an e-MH intervention (e.g. booklet version, provide extra support using computer)<br>● Consideration of the learning needs of different users (e.g. different education levels, learning disabilities) and ensure interventions are easy to understand and navigate
Innovation cost	Belief that e-MH interventions are cost-effective to the healthcare system	
	Concerns regarding user costs	

Knowledge and beliefs about the innovation[a]	Divergent views of e-MH interventions for caregivers	● Belief that e-MH can be beneficial for caregivers<br>● Concerns about e-MH interventions (e.g. impersonality) due to negative past experiences or opinions
Local attitudes	Divergent attitudes regarding caregivers and mental health	● Acknowledgement of the value of caregivers to society and people living with CKD<br>● Decreasing, but still present, stigma around mental health<br>● Viewing caregivers and their mental health needs as outside of the responsibility of kidney care providers
Local conditions	Local conditions reduce capacity/desire to support caregivers	● Caregiving role is undervalued and support for caregivers is a low priority in society<br>● Lack of services in place to support caregivers, with focus of available support on person with CKD<br>● Capacity constraints within healthcare system (e.g. not enough staff, long waitlists for mental health support)<br>● Low capacity or desire for change<br>● Poor funding for services impacts available support and makes it difficult to signpost<br>● Variable service availability by region/organisation<br>● Reduced capacity due to continued impacts from COVID-19
	Physical, digital and interpersonal environment facilitates communication and change	● Physical and digital infrastructure facilitates communication<br>● Relationships with colleagues facilitate communication, collaboration, and learning
	Increased use of technology and digital literacy in society	
Compatibility	Potential for intervention to complement or be integrated into existing practices	● Beneficial addition to existing signposting practices<br>● Good fit between the e-MH intervention and some existing healthcare delivery models (e.g. stepped care, transplant psychosocial workup)<br>● Potential for use of electronic medical record system to incorporate caregiver support and needs
	Lack of systems to support implementation	● Uncertainty regarding compatibility of such a tailored intervention in settings that work with broader populations, and a lack of systems to identify eligible caregivers<br>● Lack of systems in healthcare settings to note caregiver support needs or ensure caregivers have been referred to available services
	Potential competition with existing e-MH providers	
Mission alignment	Alignment between an organisation’s mission and the e-MH intervention varies by setting	
Access to knowledge and information	Desire for training and education about the intervention to support role in implementation	● Access to the intervention and provision of training to understand intervention content, outcomes and how the intervention works<br>● Access to contact person with more extensive knowledge of the intervention for help if needed

Need	CAREGIVER: Caregiving poses a number of challenges to the caregiver and their needs are commonly unmet	
Capability	CAREGIVER: Lack skills and knowledge which could impact caregivers' access/use of an e-MH intervention	● Low digital and/or health literacy among some caregivers<br>● Lack of knowledge of being in caregiving role
	POTENTIAL IMPLEMENTER: Ability to support caregivers is facilitated through existing relationships with caregivers and people living with CKD	
	POTENTIAL IMPLEMENTER: Lack knowledge of how to support caregivers or where to signpost to	
Opportunity	CAREGIVER: Low capacity to take part in interventions due to lack of time and/or resources	
	POTENTIAL IMPLEMENTER: Low capacity for involvement beyond endorsement	
	POTENTIAL IMPLEMENTER: Challenges navigating how to support caregiver while primary responsibility lies with the person living with CKD	● Access to caregiver may be blocked by person living with CKD making it difficult for caregiver to be provided with support<br>● Mindful of maintaining patient confidentiality
	POTENTIAL IMPLEMENTER: Regular contact with caregivers	
Motivation	CAREGIVER: Low motivation to use an e-MH intervention	● Negative views of mental health interventions<br>● Low priority of self-care
	POTENTIAL IMPLEMENTER: Empathy and understanding of caregiving experience motivates provision of support to caregivers	
Engaging - Potential implementers	Strategies needed to support awareness, engagement, and use of the intervention	● Need for easy and time-efficient intervention endorsement pathways<br>● Need to constantly work to raise awareness of the intervention, for example via word of mouth, conferences, sharing positive feedback from intervention users<br>● Need for strategies to help potential implementers recall new intervention<br>● Need for entire multi-disciplinary team to be aware of intervention to reduce missed opportunities to inform caregivers about available support
Engaging - Caregivers	Promote intervention via many pathways to reach caregivers	● Promote via healthcare settings (e.g. adverts in waiting rooms, healthcare professionals)<br>● Promote via community organisations (e.g. magazine, newsletter, email listserv)<br>● Promote via social media and through key kidney patient/caregiver advocates

[a]The main category "Knowledge and beliefs about the innovation" was added based on a construct from the original version of the CFIR. The construct was removed from the updated version of the CFIR that was used for deductive coding.

Additional table 2: Additional supporting quotes from potential implementers for each CFIR construct

CFIR construct (n = potential implementers)	Example quotes
Innovation source (n = 10)	*"If it was through the NHS then I'd know that it's obviously passed a lot of stuff to be able to get there. Which will be then reassuring from a professional point of view, that you know what you're putting your patient forward for had all of the checks and there's obviously research and evidence that it would be effective otherwise we wouldn't fund it in the first place. Through a charity, yes as long as they're an actual proper, proper charity and they're a large charity and yes, then we would happily signpost someone to that."* [P11 - mental healthcare professional]
Innovation evidence-base (n = 16)	*"If you could have some testimonials from patients, this was useful because of X, Y and Z or this is what I learnt from the course. So that's more self-report stuff. But if you've got some movement on some sort of standard measure that would be better, wouldn't it. So, stress, burden, depression, anxiety."* [P9 – kidney mental healthcare professional]<br><br>*"Personally I don't think we'd need to see evidence of that [e-MH] intervention because we're so conscious of the validity and effectiveness of - cCBT [computerised cognitive behavioural therapy] we would call it - already. So, we'd be aware that for the right groups. So, as long as there was the broad evidence base, which we know there is for cCBT. We wouldn't need to see it for an individual kind of product or module or intervention."* [P10 - mental healthcare professional]<br><br>*"I mean I don't know if I would need particularly to feel, see any medical evidence of it [the intervention]. But certainly if carers' feedback was that they felt better afterwards then, you know, that's evidence enough for me that that's kind of, you know, it's worked and it's been a positive experience for them, yes."* [P14 – professional at non-profit for caregivers]
Innovation relative advantage (n = 15)	*"[...] they wouldn't have to have the courage to come up with it. It would be an easy thing to just say,' look, this exists' rather than someone having to sit there and say, 'look, is there anything for me, I'm really struggling'. It's something they [the carers] can engage with outside of the clinic environment"* [P1 – kidney healthcare professional]<br><br>*"Yes, I think because you can offer it out to everybody and anybody almost, so you've got that facility. Whereas, were only with very small staff numbers, even though we've got lower patient numbers. We couldn't offer it to every single person, we couldn't go out knocking and canvassing opinions on it."* [P9 – kidney mental healthcare professional]<br><br>*"And potentially [feel] less guilt because they could look at it [the e-MH intervention] when the person they're caring for is asleep or is settled or whatever the situation is."* [P11 – mental healthcare professional]<br><br>*"Things that people can dip in and dip out of and recordings are really useful because people sometimes like to watch them again."* [P13 – professional at non-profit for caregivers]
Innovation design (n = 18)	*"As I said, only just the, the big words, the keeping it simple, keeping it at a level that people are going to understand, because we obviously have a mixture of education and non-educated people that will be accessing it."* [P4 – kidney healthcare professional]<br><br>*"So we send people to [Talking Therapies] and they come back, and people say well when are you going to be able to stop doing dialysis then? When are your kidneys going to get*

	better? Just stuff like that really is a bit of a relationship killer. [...] So, I do think knowledge about renal is important and a baseline knowledge about the impacts of mental health." [P9 – kidney mental healthcare professional]<br><br>*"Maybe even just, well this could be a good and a bad I can imagine [...]. So like having a reminder to their phone to do it [to use the intervention] because [...] when you're so busy, that another day would go past and, oh God I didn't access it or something like that. And another day passes, oh God I forgot to access it again. So maybe just having a reminder"* [P11 – mental healthcare professional]<br><br>*"I like the idea of an app. I like the idea of the online resources. I thought it was good to think that there'll be somebody there that would do a bit of regular contact, for that six weeks, because I think that would be really great."* [P13 - professional at non-profit for caregivers]
Innovation cost (n = 8)	*"I suppose the only thing that would maybe make it difficult is if there was a cost involved to the carer because then we'd be selling something and that's not something we normally do. [...] So I think if there was a cost implication that would make it difficult for us to, without working something out about, you know, tear this voucher out and you get in for half price or something like that. But yes, I think that would be maybe difficult."* [P14 - professional at non-profit for caregivers]
Knowledge and beliefs about the innovation (n = 18)	*"I think it could be really positive. Yes, they [carers] are a neglected group. As you said earlier, it depends on how engaged a clinician is to whether they're even involved in a consultation or ignored or asked to leave or whatever. So I think having a resource that's dedicated to them is almost an acknowledgement of their importance. Even just saying, look, we've got this resource that exists suggests that other people have struggled or other people have found it difficult or found they need more information or whatever. I think even just having it existing gives them some validation for what they may be feeling."* [P1 – kidney healthcare professional]<br><br>*"I think that [the intervention] would be helpful obviously on a practical level, particularly. But also it may give them more of a feeling of togetherness with other people in the same situation. Like they know that other people who are looking after people with chronic kidney disease, conditions, whatever are potentially all experiencing very similar things [...]"* [P11 – mental healthcare professional]<br><br>*"[...] I don't like it [e-mental health] on a sort of personal level. I would rather be speaking to you in person now than I would via Zoom. Notwithstanding it has its place [but] you know, our interaction would be much richer. We'd have more depth. We'd have more meaning if we were sitting in a room together. We would get to know each other better just because [...] we're in a room together and all those unspoken signals and stuff. And to me that's really important when it comes to mental health of course. You know, it's so important. And so I wonder what would be lacking. [...] you want someone to put their arms around you, you want someone to, metaphorically speaking, you want some to be with you, you want someone on that journey. And I don't think, pressing a leave button at the end of [a session] is caring enough."* [P15 - professional at non-profit for caregivers]
Local attitudes (n = 15)	*"[...] some people [kidney healthcare professionals] won't even ask about emotional wellbeing or a carer might say 'I'm really struggling, because we haven't got any benefits, or I can't work anymore'. The doctor will just go, 'I'm really sorry to hear that', but that's it, that's left, isn't it. Because there's not always seen as their remit, and they haven't got time to do anything about it."* [P9 – kidney mental healthcare professional]

	"I think also probably in general, it's often remarked that people are more open to talking about mental health these days, than they were in years or decades previously. So that generally greater level of awareness probably is useful in helping carers to open up and think about their own mental health and maybe seek support if they are struggling." [P16 - professional at non-profit for caregivers]
Local conditions (n = 18)	*"I think from what I've been hearing recently it's waiting lists. I'm just trying to think who I was talking to yesterday. I was talking to a carer yesterday who desperately needs some psychological support for her son who she's caring for but for herself as well. She feels like they're both really struggling. And they just can't get into the system at the moment. So they've been told that there'll be a long wait before they can get any support. So, yes, that's quite a worry I think."* [P2 – kidney healthcare professional]<br><br>*"The internet's a big thing these days, it's part of life. You see children of two with tablets these days. So yes, it's a big part of life and it's going to be an even bigger part of the next generations. So definitely I think E-learning is something out there"* [P4 – kidney healthcare professional]<br><br>*"We kind of create our own little directory [of support services to refer people to] that obviously goes out of date about five minutes after we've put it together because there's always new groups, support groups and stuff starting, especially then with Covid everything went out the window. And people lose their funding and things so it makes it tricky [to refer people to support]."* [P11 – mental healthcare professional]<br><br>*"We know from our research that many of the services that both carers and the people that they [care for] are unfortunately still heavily disrupted after the pandemic. So many services for carers, services for disabled people, anything else in the community, things like respite services or day services, care homes, blah blah, blah. Many of these services of course were massively disrupted during the lockdowns and the coronavirus restrictions. But even though we're about two and a half years on from that, we're picking up from our research that there's still quite a significant disruption. Many of these services haven't fully reopened and of course it's made even harder, because of the well-known shortages of care workers and things. They're really struggling to get the staff that they need. So, I guess one thing to just be aware of, is that unfortunately the overall level of services in the community for mental health or for carers in general or whatever, is unfortunately still quite low and hasn't fully recovered from the pandemic."* [P16 - professional at non-profit for caregivers]<br><br>***"INT:*** *Okay. And to what extent are new ideas embraced and used to make improvements?* ***RES:*** *All of the time. All of the time. […] Yes, all of the time. And we're always learning, always trying to evolve and implement new projects and new services which is why time resources and funding is key to everything that we do, yes."* [P17 – professional at kidney specific non-profit organisation]
Compatibility (n = 16)	*"It [the e-mental health intervention] could be easily fitted in without taking any more time. I think if anything it would make things, it would speed things up because you'd have, instantly know what to say, how to signpost them correctly without it just relying on that healthcare professional's knowledge and confidence, you know, that it's done correctly really."* [P6 – kidney healthcare professional]<br><br>*"I wonder if because we've already got SilverCloud it would be something that, well we've got our e-learning through that organisation and we've used them for a long time and it's worked quite well. That we would expect it to come through that provider, because we've already got that relationship."* [P10 - mental healthcare professional]

Mission alignment (n = 6)	*"Well we've got that target [that] we have to always improve access. So we are always looking for new client groups that we can work in and you know carers could be a good one, you know, to be the next one down the line. So that, you know, that could be a definite opportunity because we do have access targets as well."* [P12 – mental healthcare professional]<br><br>*"I think it's just because they [staff working at the organisation] can see that there would be a real positive impact on carers' lives, you know. I mean that's the whole aim of our organisation is to help and support carers. And anything that we think would make a real difference to their lives then, you know, that's what we would go for if we can, you know, if we can get the funding for it then we would go for it, yes."* [P14 – professional at non-profit for caregivers]<br><br>*"I think we all have a strong sense of mission, even when we're all delivering lots of different activities. That's one of the good things about the culture of the third sector. I guess, because of that any new intervention like an app, if we think it's going to help the mission of supporting unpaid carers, if it ties into our overarching goal, then we'll probably be quite enthusiastic."* [P16 - professional at non-profit for caregivers]
Access to knowledge and information (n = 13)	*"I think I'd like to see how it worked, I'd like to try it myself and so that I could talk someone through it. And then be able to say to them, look you know- Or have some feedback yourself with your team to say how it worked"* [P3 – kidney healthcare professional]<br><br>*"I suppose we would want to know what it [the e-mental health intervention] involves [...] maybe like a summary of what the [...] caregiver would experience by accessing it. What things they might need to do. Timeframes. You know, how often they need to interact with it. Or if there's an expectation from them versus [...] it's just there as and when they need it."* [P6 – kidney healthcare professional]<br><br>*"From the start, so it would be you know, if it was an established programme then you would want the person coming along to demonstrate how it [the intervention] all works and what's the benefits of it."* [P13 – professional at kidney specific non-profit organisation]
Need (n = 15)	*"As I said, it's a massive responsibility, which people don't always realise. They just kind of crack on with it and they don't really [recognise] that it is affecting their mental health and their own stress levels and anxiety."* [P2 – kidney healthcare professional]<br><br>*"[...] an informal carer is the supreme being of caring with no boundaries, no training, no clock off, no support team, [no] network of staff to moan and bitch about what happened today. And you know, and 'oh do you mind doing that because I'm just doing this'. You know, there's nobody else to do it. So they're often, you know, they don't get a holiday and a break and a big pay packet at the end of it. So they're frazzled, their tired, they haven't often taken care of themselves so they're physically not at their optimum."* [P12 – mental healthcare professional]
Capability (n = 15)	*"But I think before maybe there was a feeling that if you did find out that the caregiver was really struggling, what would you do? [...] So yes, I guess that's probably another issue as well."* [P1 – kidney healthcare professional]<br><br>*"I think the younger generations may find it helpful because they're more in tune with that sort of thing. They view their lives electronically. The older ones maybe won't feel confident with a device to navigate it and that might put them off."* [P3– kidney healthcare professional]

	"Then the other probably most significant point is that, we know from our research that many people who start looking after someone, they often take a very long time to actually see themselves as an unpaid carer. They just think, 'well, I'm just a parent maybe looking after a disabled child' or 'I'm just looking after my spouse who is very ill at the moment'. If it takes people, as it often does, months or even years to start to think of themselves as a carer. Then it means that they're less likely to be engaging with services to support carers, with their mental health for example." [P16 - professional at non-profit for caregivers]
Opportunity (n = 17)	*"I think the problem you would have is if it impacted on their [staff's] workload a lot, if it was them. Them having to give up an hour or two or three a week of their normal daily working time to implement it or facilitate it. That might be where you have the problem, but I think as sending out information, signposting thing most people would embrace it I would think."* [P2 – kidney healthcare professional]<br><br>*"And then financially as well people might say 'well actually I don't use the internet because I don't, I can't afford it', especially with the way things are going at the moment."* [P5 - kidney healthcare professional]<br><br>*"I think probably a time factor, the guy I'm thinking of supporting someone with kidney disease. He's running around doing everything for four primary school children and looking after his wife. [...] He would himself struggle to attend appointments [for his mental health] or see the point in doing so probably."* [P10 – mental healthcare professional]<br><br>*"[...] you'd be surprised how many partners ring up [for support] when there's a, 'I've rung up because my husband's gone to dialysis, he doesn't like me talking to others'."* [P18 – professional at kidney specific non-profit organisation]
Motivation (n = 13)	*"Whether people do it, so I think sometimes people, it's a bit of self-care isn't it, I guess. People who are already caring for other people are not necessarily very good at caring for themselves. I wonder if it would just slip down the bottom of a to do list of, 'oh I'll look at that resource at some point'."* [P1 – kidney healthcare professional]<br><br>*"A bit of a barrier to maybe accessing treatment because of kind of maybe slight assumptions about what mental health support means and just reluctant to refer themselves, accept help or even think that things could be any different."* [P10 - mental healthcare professional]
Engaging - Potential implementers (n = 16)	*"So, I think if this intervention was to be embedded in routine hospital care or made available as routine in hospital care, everyone would have to know about it. Yes, I think because people often discuss their transplant concerns with their peritoneal dialysis nurse or their haemodialysis concerns with their anaemia nurse. So, I think being ready and knowing about the availability of resources for everyone involved in the kidney team is really important. Surgeons for example often get asked about stuff when they're seeing, you know, maybe because surgeons are thought of knowing answers to lots of things. Yes, I think if the resource is embedded it would have to be something that everyone was aware of, because otherwise they'll be missed opportunities for it to be used."* [P1 – kidney healthcare professional]<br><br>*"If it was very easy, tick box, quick referral, they'll love it. So tick that box, patient referred, I don't have to do anything else, yes. We'll all like that."* [P3 – kidney healthcare professional]<br><br>*"Just as long as you had a clear pathway, you know, with the right element of referral. If there's a very simple referral form maybe, that's, you know something like that, but very simple. Not complex because we have plenty of them. Just easy, make it easy. Please make it easy. That's it."*

	[P15 – professional at non-profit for caregivers]
Engaging – Caregivers (n = 9)	*"I think it being available or advertised through a hospital would be the best, I guess in my mind. I guess by hospital I mean everything that goes with that hospital, so the hospital and its satellite dialysis unit, the hospital and its outpatient clinics."* [P1 – kidney healthcare professional]<br><br>*"But I think when patients go to their clinic appointments for, you know, their kidney health, at that point those people should be looking into the carers because although we [staff in NHS Talking Therapies] could signpost them in our service, it would be hit and miss because they'd have to access us in the first place. Whereas if you catch them at that stage then you're more likely to see them than we are."* [P11 – mental healthcare professional]<br><br>*"So another way would be through our social media accounts. Because obviously like any other carer organisation and various other charities, we have our social media accounts. Many of them will be followed. Most of our followers are probably carers. Many of our other followers are professionals that work with carers, maybe in the health service or in councils, people who want to keep up to date with these sorts of issues. So social media might be a way."* [P16 - professional at non-profit for caregivers]
